# Distinct Actions of Akt1 on Skeletal Architecture and Function

**DOI:** 10.1371/journal.pone.0093040

**Published:** 2014-03-24

**Authors:** Aditi Mukherjee, Emily A. Larson, Robert F. Klein, Peter Rotwein

**Affiliations:** 1 Department of Biochemistry and Molecular Biology, Oregon Health & Science University, Portland, Oregon, United States of America; 2 Bone and Mineral Research Unit, Department of Medicine, Oregon Health & Science University, Portland, Oregon, United States of America; 3 Portland Veterans Affairs Medical Center, Oregon Health & Science University, Portland, Oregon, United States of America; INSERM U1059/LBTO, Université Jean Monnet, France

## Abstract

Skeletal integrity is dependent on the coordinated actions of bone-forming osteoblasts and bone-resorbing osteoclasts, which recognize and respond to multiple environmental inputs. Here we have studied the roles in bone development and growth of Akt1 and Akt2, two closely related signaling proteins, by evaluating mice lacking either of these enzymes. Global deficiency of Akt1 but not Akt2 caused a reduction in whole body and femoral bone mineral density, in femoral cortical thickness and volume, and in trabecular thickness in both males and females when measured at 20-weeks of age, which was reflected in diminished femoral resistance to fracture. Haplo-deficiency of Akt1 in male mice also decreased femoral cortical and trabecular skeletal parameters, and reduced bone strength. Cell-based studies showed that genetic Akt1 deficiency diminished the rate of proliferation of osteoblast progenitors and impaired osteoclast differentiation in primary culture but that loss of Akt2 did not. Our results demonstrate differential effects of Akt1 and Akt2 on skeletal maturation and architecture through actions on both osteoblast and osteoclast precursors.

## Introduction

Bone growth during childhood and bone remodeling in the adult are complex processes that promote and sustain skeletal integrity, mass, and strength. Bone remodeling in the adult skeleton consists of two temporally and spatially coupled phases, resorption by osteoclasts, and new bone deposition by osteoblasts [1]–[5]. Both stages of remodeling are controlled by interactions between local and systemically-derived signals mediated by mechanical strain, cytokines, growth factors, and hormones, and genetically-determined programs regulated by bone cell-specific transcription factors [2], [3], [5].

The most common bone disease in adults is osteoporosis, which is characterized by low bone mineral density (BMD) and structural deterioration of the skeleton, leading to increased fragility and enhanced risk of fractures. BMD, which represents the best clinical predictor of future osteoporotic fracture risk [6], [7], is a complex trait that also is controlled by the interplay of multiple environmental factors with many genetic influences, each with individually small effects [8]. Although recent reports have begun to identify some potential genetic determinants of BMD and bone strength in humans [9], resolving this problem will be a difficult challenge because of the heterogeneity of human populations. One approach to gain insights into genetic influences on the skeleton has been to exploit animal model systems to identify possible candidate genes for more focused human investigation [10]–[12]. Although no animal model can duplicate all aspects of human osteoporosis, objective evaluation in other species of the impact of individual genes on specific traits, such as BMD or bone strength, can be useful for subsequent study of their potential contributions to disease susceptibility in humans.

Multiple signaling pathways activated by different cytokines, hormones, and growth factors influence bone mass and strength by actions on osteoblasts and osteoclasts and their progenitors [3], [5]. Among these many signaling cascades, there is evidence that the PI3-kinase - Akt pathway is critical for bone development, growth, and skeletal integrity [13]–[17]. Mice globally lacking Akt1 and Akt2, two of the three Akt paralogs in mammals [18], have a severe bone deficiency phenotype at birth along with major deficits in other growth and developmental programs [17]. Blocking the PI3-kinase - Akt pathway also has been shown to impair longitudinal bone growth in short-term organ culture, and to prevent osteoblast differentiation [19]. Conversely, loss of the phosphatase Pten in osteoblast precursors, leading to increased Akt signaling [20], [21], has been found to cause enhanced bone formation and increased bone mass in mice [22]. Here we have directly assessed the role of individual Akts in bone development and growth in mice. We find that global deficiency of Akt1 but not Akt2 caused a decline in whole body and long bone BMD, which was reflected in diminished femoral resistance to fracture. Haplo-deficiency of Akt1 in male mice also reduced femoral BMD and bone strength. At least part of the impact of lack of Akt1 on bone could be attributed diminished proliferation of osteoblast precursors and to reduced differentiation of osteoclast progenitors. Our results identify functional differences between the actions of Akt1 and Akt2 in the skeleton.

## Materials and Methods

### Reagents

Alpha Minimal Essential Medium (α-MEM), Dubecco's Modified Eagle's Medium (DMEM), fetal calf serum, trypsin/EDTA solution, phosphate-buffered saline (PBS), penicillin-streptomycin, and Superscript III first-strand synthesis kit were purchased from Life Technologies (Carlsbad, CA). Okadaic acid, alizarin red, sodium orthovanadate, TRAP staining kit, ascorbic acid, and β-glycerol phosphate were from Sigma-Aldrich (St. Louis, MO). Protease inhibitor and NBT/BCIP tablets were from Roche Applied Sciences (Indianapolis, IN). Polymerases were purchased from Roche Applied Sciences, and Fermentas (Hanover, MD). AquaBlock EIA/WIB solution was from East Coast Biologicals (North Berwick, ME), and the BCA protein assay kit was obtained from Pierce Biotechnologies (Rockford, IL). Immobilon-FL was from Millipore Corporation (Billerico, MA). RANKL and m-CSF were purchased from R&D systems (Minneapolis, MN). Production and use of recombinant BMP2 has been described [23]. Antibodies were obtained from the following vendors: anti-Akt1 (AbCam, Cambridge, United Kingdom), anti-Akt, anti-phospho-Akt (Ser^473^), and anti-Akt2 (Cell Signaling Technology, Beverly, MA), anti-α-tubulin, Sigma Aldrich (St. Louis, MO), anti-Runx2 (Santa Cruz Biotechnology, Santa Cruz, CA). Goat-anti-rabbit IgG-IR800 and goat anti-mouse IgG-IR680 were from Rockland Immunochemical (Gilbertsville, PA). Other chemicals and reagents were purchased from commercial suppliers.

### Ethics statement

All animal procedures were approved by the OSHU and Portland VA Institutional Animal Care and Use Committees (protocol number IS00001929; institutional IACUC assurance number A3304-01), and were performed in strict accordance with National Institute of Health guidelines for the care and use of animals in research.

### Animals

Heterozygous Akt1 and Akt2 deficient mice bred onto the C57BL/6 background were obtained from Dr. Morris Birnbaum (University of Pennsylvania). All mice used in experiments were maintained under identical conditions at the Portland VA Veterinary Medical Unit. Heterozygous littermates were crossed to generate wild type (WT), Akt homozygous null, and Akt heterozygous mice for study. Genotyping was performed by PCR on tail DNA, as described [24]. Mice used for breeding were maintained for no more than 3 generations. After weaning, mice were housed in groups of 2–5 animals per cage, and were provided with rodent chow (Diet 5001, PMI Feeds, Inc., St. Louis, MO) and water *ad libitum*, and were maintained in a 12 hr light-dark cycle at 21±2°C. Mice were euthanized by CO_2_ inhalation, and weighed to the nearest 0.1 g. The femurs were harvested immediately from each mouse used for bone measurements, were wrapped in sterile gauze soaked in PBS, and stored at −20°C until subsequent analyses.

### Skeletal phenotyping

Whole body and femoral bone mineral measurements were determined by peripheral dual energy X-ray absorptiometry (pDXA; PIXImus, GE-Lunar, Madison, WI, USA). Routine calibration was performed daily with a defined standard (phantom). Cortical femoral shaft bone geometry was examined with a desktop x-ray microtomographic scanner (SkyScan Model 1074, Aartselaar, Belgium). Images were analyzed with Optimas software (version 6.2; Media Cybernetics, Silver Spring, MD). The three-dimensional organization of femoral cortical and distal metaphyseal bone was evaluated with data collected with a Scanco medical MicroCT 35 (Brüttisellen, Switzerland), using a 70 kVp X-ray source, and analyzed with system software, with data assessments being performed as outlined in Bouxsein et al [25]. The threshold for analysis was determined empirically and set at 245 (scale 0–1000). For femoral cortical bone, 18 slices were taken at femoral mid-shaft (totaling 216 μm). Measurements included cross-sectional volume, cortical volume, marrow volume, cortical porosity, and cortical thickness. For the femoral metaphysis, 40 slices of secondary spongiosa (totaling 480 μm) were evaluated. Trabecular number, trabecular thickness, trabecular spacing, and bone volume/total volume (BV/TV) were assessed. To determine femoral strength, femurs of 20-week old mice were tested to failure by three-point bending on a high-resolution materials test apparatus (Model 4442, Instron Corp., Canton, MA). Load and displacement data were recorded and failure load was determined using system software.

### Osteoblast growth and differentiation

Neonatal mouse calvaria and primary bone marrow stromal cells (MSCs) from ∼12 week old male mice were isolated and grown in serum-containing medium, as described [26]. Cell growth was measured by counting trypsinized cells at daily intervals with a hemocytometer. Confluent cells were incubated in osteogenic medium (OM: DMEM, 10% fetal calf serum, 50 μg/mL ascorbic acid, 10 mM β-glycerol phosphate) supplemented with recombinant BMP2 [200 ng/mL]. OM with BMP2 was replaced every 48 hr. Staining for alkaline phosphatase and Alizarin red was performed as described [23], using cells fixed with 70% ethanol. For detection of mineralization, cells were fixed in 70% ethanol for 10 min, and stained with 2% Alizarin red solution (pH 4.1–4.5) for 1 min at 20°C. Images were captured and analyzed with the LiCoR Odyssey Infrared Imaging System, using software version 3.0 (LiCoR, Lincoln, NE).

### Osteoclast differentiation

Bone marrow stromal cells were isolated from 12 week old mice and enriched for marrow macrophages by Ficoll gradient centrifugation [27]. For osteoclast differentiation, cells were incubated α-MEM with 10% fetal calf serum containing m-CSF [100 ng/mL] and RANKL [35 ng/mL]. Medium was changed every 48 hr. Osteoclasts were stained for expression of tartrate resistant acid phosphatase (TRAP), as described [27] after fixation with 70% ethanol by incubation with Fast Garnet and tartrate solution for 1 hr at 37°C, followed by 3 washes with distilled water. Counterstaining was performed with hematoxylin dye, and images were captured using a Nikon Eclipse E800 compound microscope with CCD camera. Image J software was used to quantify TRAP positive area.

### Analysis of gene expression

Whole cell RNA was extracted and reverse transcribed (2 μg) with the Superscript III first-strand cDNA synthesis kit using oligo (dT) primers [28]. PCR was performed with 20 ng of osteoblast cDNA in 1 μL, as described [23], [24], [28], and previously published primer pairs for mouse Akt1, Akt2, Runx2, osteocalcin, and S17 [23], [24], [28]. Cycle numbers were within the linear range for each primer pair, and ranged from 20–30 cycles. Results were visualized after agarose gel electrophoresis.

### Protein extraction and immunoblotting

Whole cell protein lysates were prepared as described [28], and aliquots were stored at −80°C until use. Protein samples (10–30 μg/lane) were resolved by SDS-PAGE (10% separating gel) and transferred to Immobilon-FL membranes. After blocking with 25–50% AquaBlock solution for 1 hr at 20°C, membranes were incubated sequentially with primary and secondary antibodies [28]. Primary antibodies were used at dilutions ranging from 1∶1000–1∶15,000, and secondary antibodies at 1∶5000. Detection was by chemi-fluorescence. Data were captured using the LiCoR Odyssey Infrared Imaging System, using software version 3.0 (LiCoR, Lincoln, NE).

### Statistical Analysis

**Figure 1:**
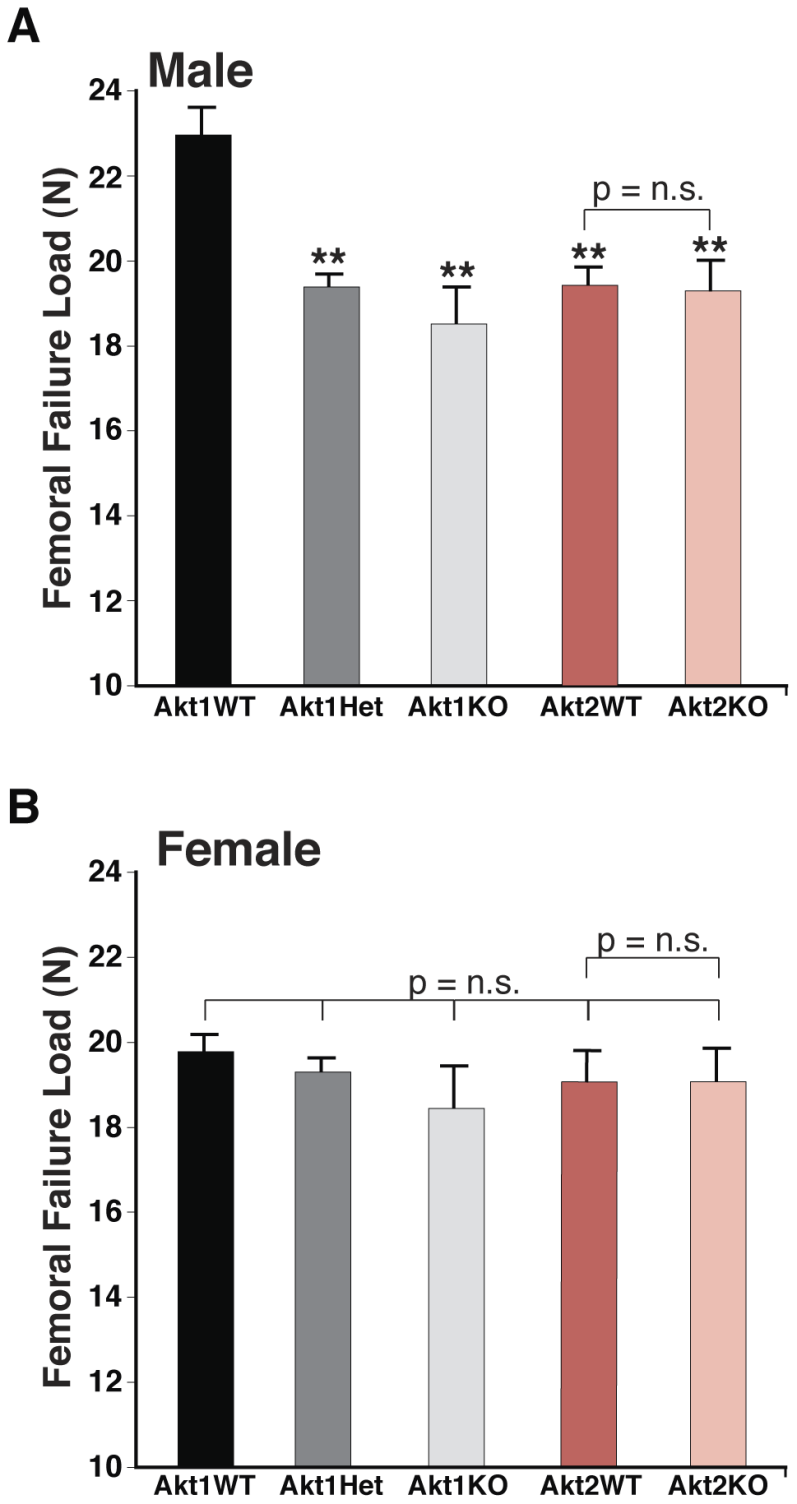
Reduced bone strength in femurs from heterozygous and homozygous Akt1 deficient male mice. Femoral fracture load was measured in isolated femurs from 20-week old male and female mice using a high-resolution materials test apparatus. **A**. Results in male mice (mean ± SEM; **- p≤0.001 vs. Akt1 WT, n = 10–12/genotype). **B**. Results in female mice (n = 5–10/genotype; p =  not significant (n.s.) for all comparisons).

**Figure 2:**
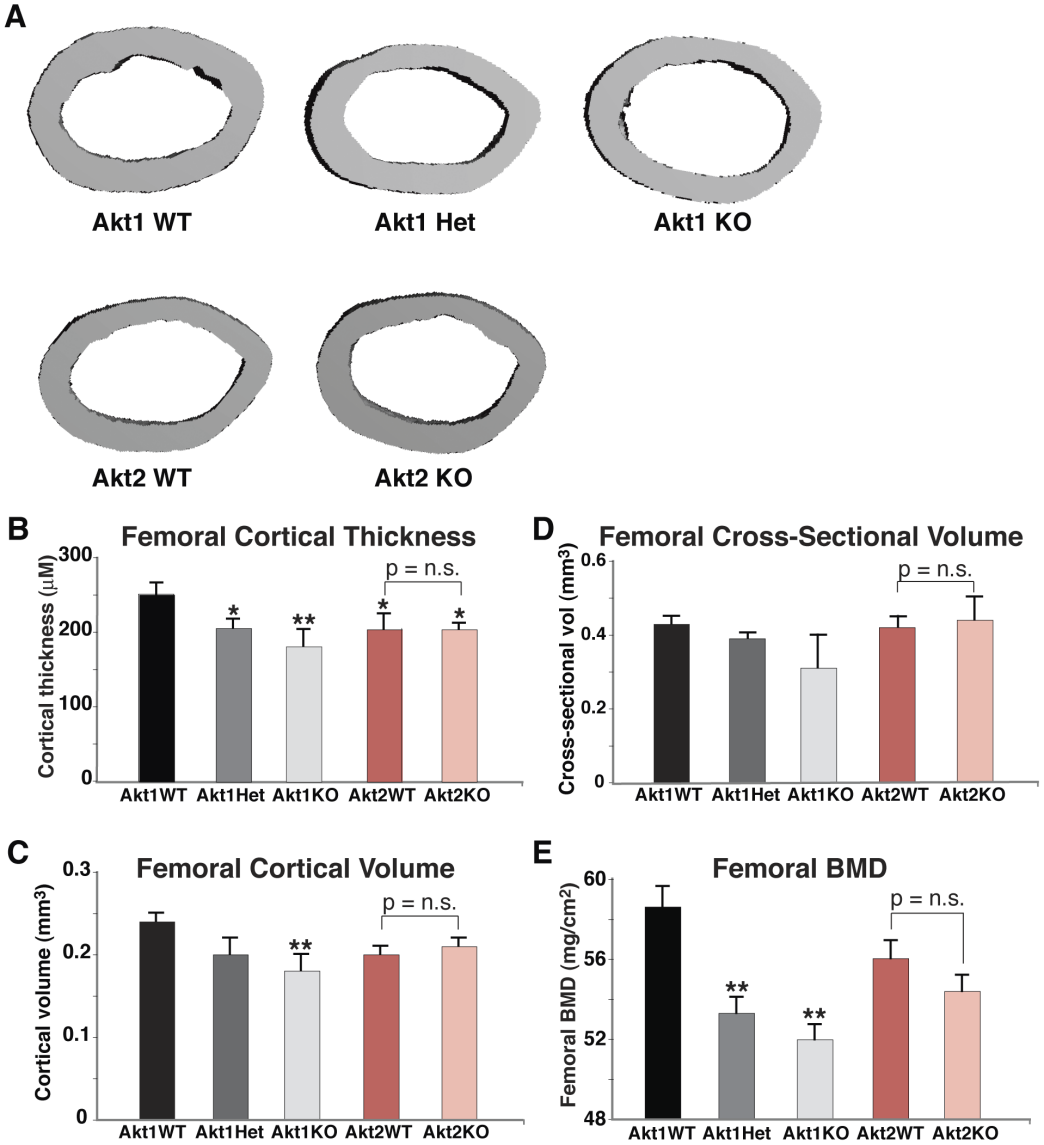
Diminished cortical bone mass in heterozygous and homozygous Akt1 deficient male mice. **A**. Representative micro-CT images of a cross-section of mid-shaft femurs from male 20-week old mice with the indicated genotypes. **B–E**. Graphical presentation of data from male mice for each indicated genotype (mean ± SEM, *- p≤0.01, **- p≤0.001 vs. Akt1 WT). **B**. Femoral cortical thickness (n = 4–5/genotype). **C**. Femoral cortical volume (n = 4–5/genotype). **D**. Femoral cross-sectional volume (n = 4–5/genotype). **E**. Femoral BMD (n = 12–19/genotype).

**Figure 3:**
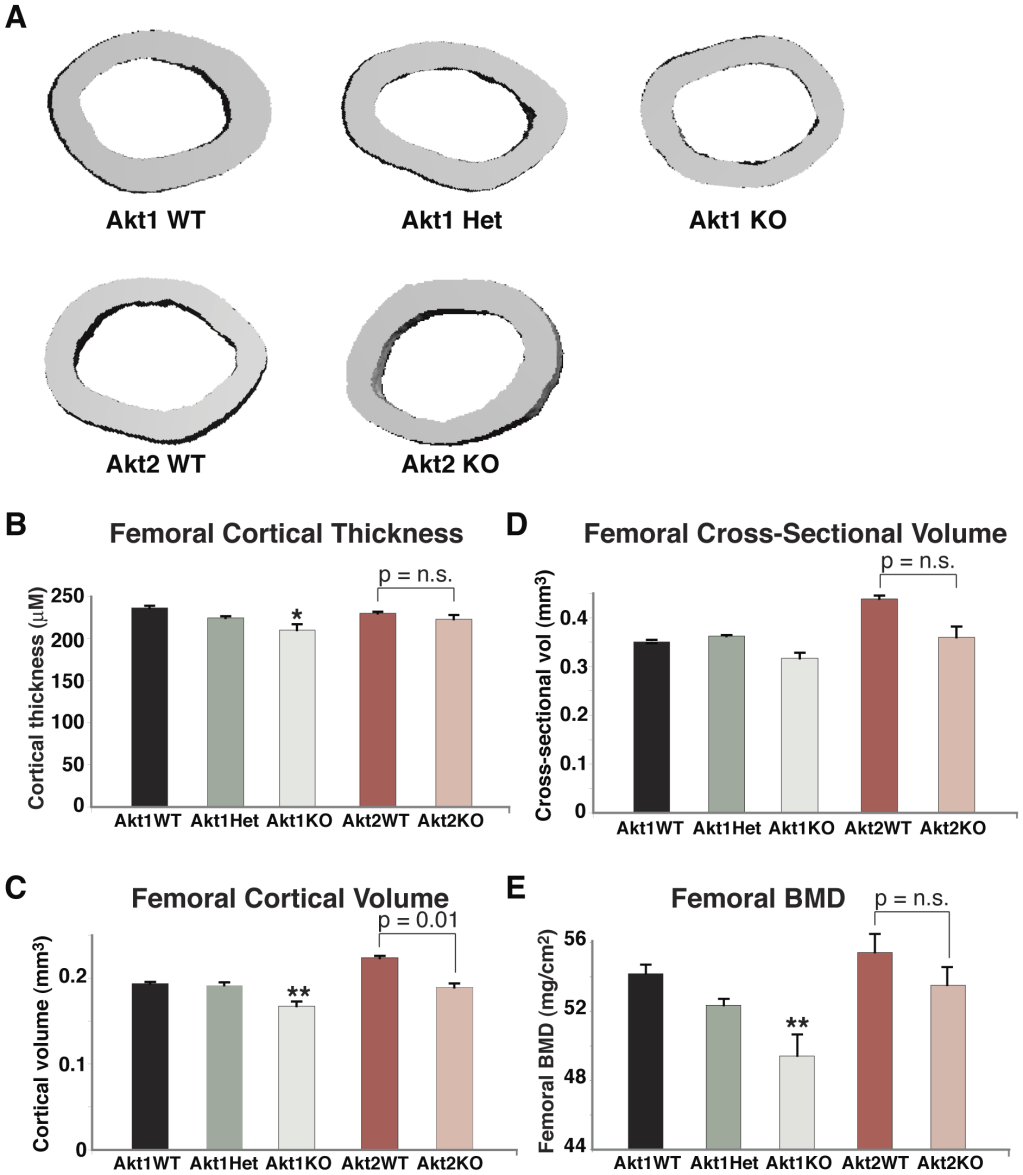
Minimal alteration in cortical bone mass in Akt1 or Akt2 deficient female mice. **A**. Representative micro-CT images of a cross-section of mid-shaft femurs of female 20-week old mice with the indicated genotypes. **B–E**. Graphical presentation of data from female mice for each indicated genotype (mean ± SEM, *- p≤0.01, **- p≤0.001 vs. Akt1 WT). **B**. Femoral cortical thickness (n = 4–5/genotype). **C**. Femoral cortical volume (n = 4–5/genotype). **D**. Femoral cross-sectional volume (n = 4–5/genotype). **E**. Femoral BMD (n = 12–19/genotype).

**Figure 4:**
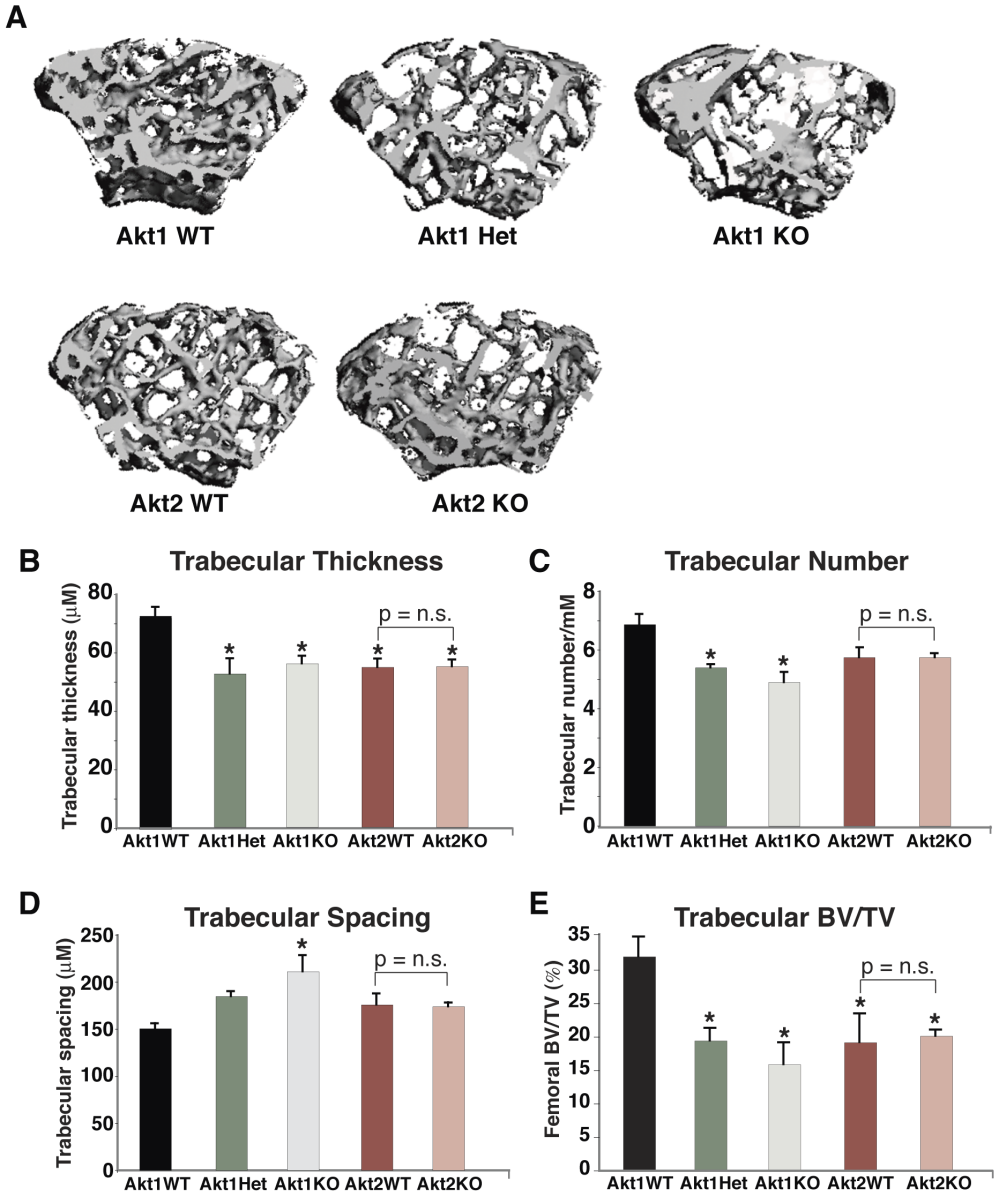
Reduced trabecular bone mass in heterozygous and homozygous Akt1 deficient male mice. **A**. Representative micro-CT images of the distal femoral metaphysis of male 20-week old mice with the indicated genotypes. **B–E**. Graphical presentation of data from 4–5 male mice for each indicated genotype (mean ± SEM; *- p≤0.01 vs. Akt1 WT). **B**. Trabecular thickness. **C**. Trabecular number. **D**. Trabecular spacing. E. BV/TV. Other statistical data are indicated.

**Figure 5:**
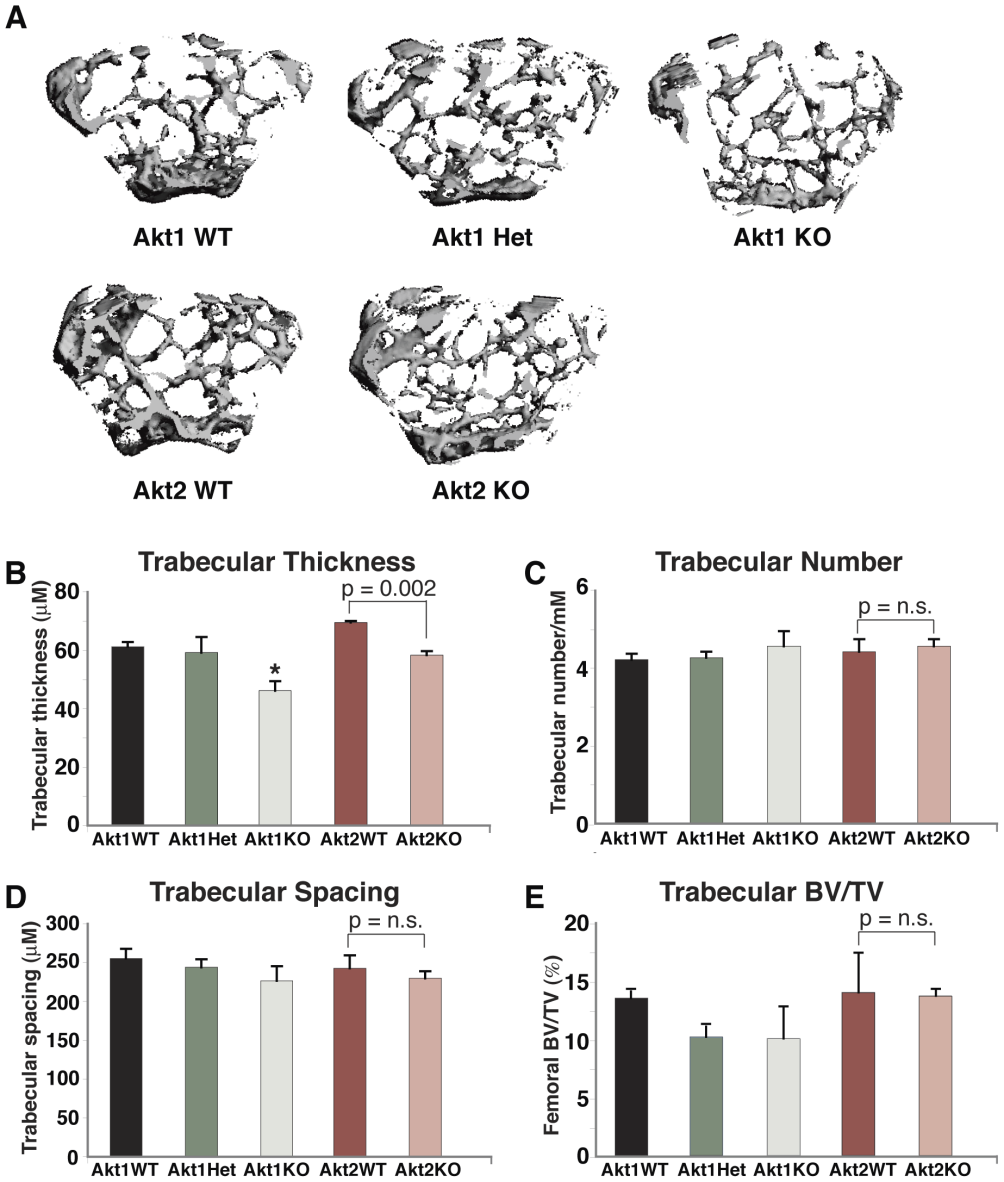
Minimally reduced trabecular thickness in Akt1 deficient female mice. **A**. Representative micro-CT images of the distal femoral metaphysis of female 20-week old mice with the indicated genotypes. **B–E**. Graphical presentation of data from 3–4 female mice for each indicated genotype (mean ± SEM). **B**. Trabecular thickness (*- p≤0.01 vs. Akt1 WT). **C**. Trabecular number. **D**. Trabecular spacing. E. BV/TV. Other statistical data are indicated.

**Figure 6:**
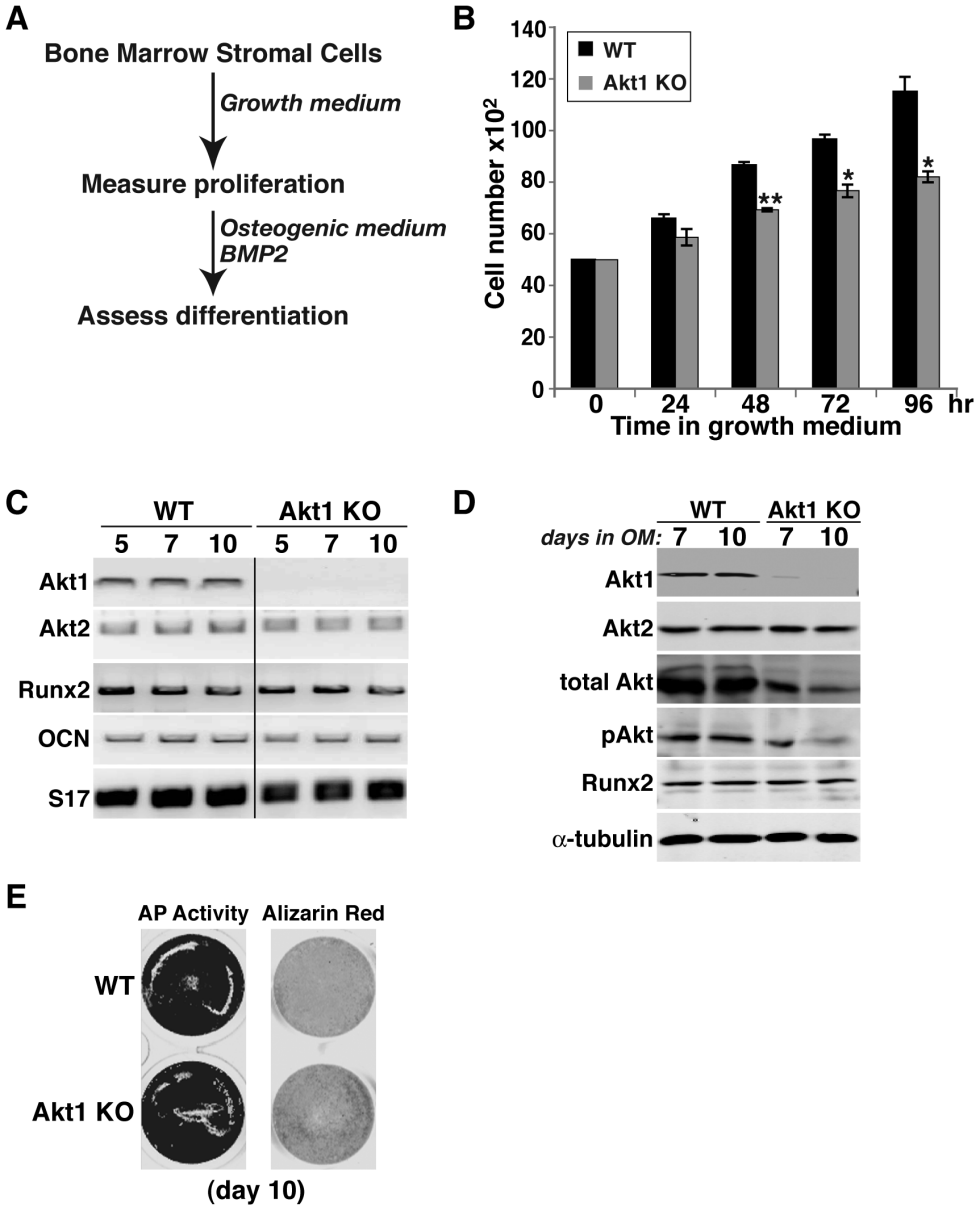
Reduced proliferation but normal osteogenic differentiation of bone marrow stromal cells from Akt1 deficient male mice. **A**. Experimental scheme: bone marrow stromal cells from 20-week old WT and Akt1 KO mice were incubated in growth medium for 4 days, and then in osteogenic medium (OM) plus 200 ng/ml BMP2 for up to 10 days. **B**. Measurement of cell number by daily counting (mean ± S.D., n = 3 experiments; *- p<0.01, **- p<0.003 vs. Akt1 WT). **C**. Measurement of gene expression by RT-PCR on days 5, 7, and 10 after incubation in OM plus BMP2 for Akt1, Akt2, Runx2, osteocalcin (OCN), and S17 mRNAs. **D**. Immunoblots of whole cell protein lysates on days 7 and 10 after incubation in OM plus BMP2 for Akt1, Akt2, total Akt, phospho (p) Akt, Runx2, and α-tubulin. **E**. Alkaline phosphatase (AP) and Alizarin red staining on day 10.

**Figure 7:**
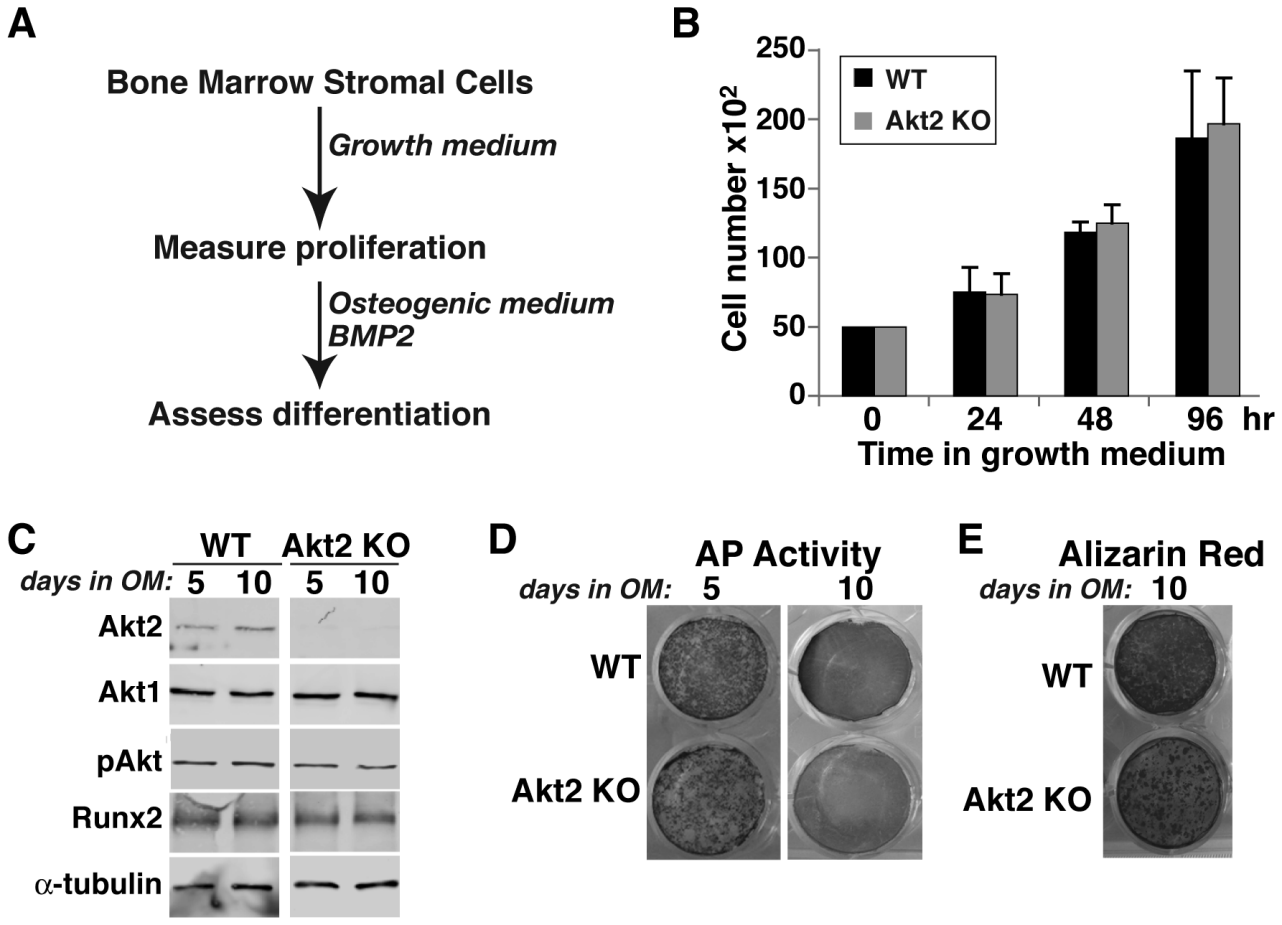
Normal proliferation and osteogenic differentiation of bone marrow stromal cells from Akt2 deficient mice. **A**. Experimental scheme: bone marrow stromal cells from 20-week old WT and Akt2 KO mice were incubated in growth medium for 4 days, and then in OM plus 200 ng/ml BMP2 for up to 10 days. **B**. Measurement of cell number by daily counting. **C**. Immunoblots of whole cell protein lysates on days 5 and 10 after incubation in OM plus BMP2 for Akt1, Akt2, total Akt, pAkt, Runx2, and α-tubulin. **D**. Alkaline phosphatase (AP) activity on days 5 and 10. **E**. Alizarin red staining on day 10.

**Figure 8:**
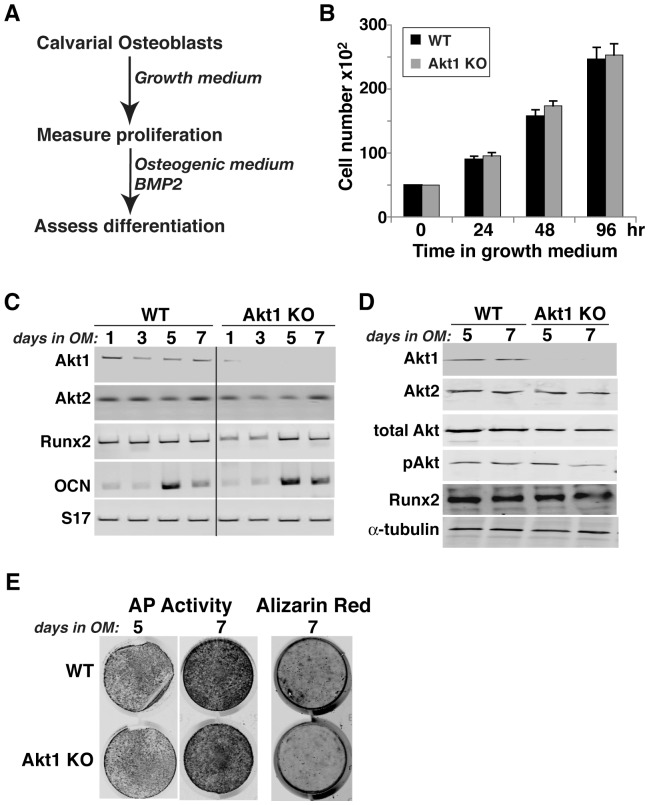
Normal proliferation and differentiation of calvarial osteoblasts from Akt1 deficient mice. **A**. Experimental scheme: calvarial cells from newborn WT and Akt1 KO mice were incubated in growth medium for 4 days, and then in osteogenic medium (OM) plus 200 ng/ml BMP2 for up to 7 days. **B**. Measurement of cell number by daily counting. **C**. Measurement of gene expression by RT-PCR on days 1, 3, 5, and 7 after incubation in OM plus BMP2 for Akt1, Akt2, Runx2, OCN, and S17 mRNAs. **D**. Immunoblots of whole cell protein lysates on days 7 and 10 after incubation in osteogenic medium (OM) plus BMP2 for Akt1, Akt2, total Akt, pAkt, Runx2, and α-tubulin. **E**. Alkaline phosphatase (AP) activity on days 5 and 7, and Alizarin red staining on day 7.

**Figure 9:**
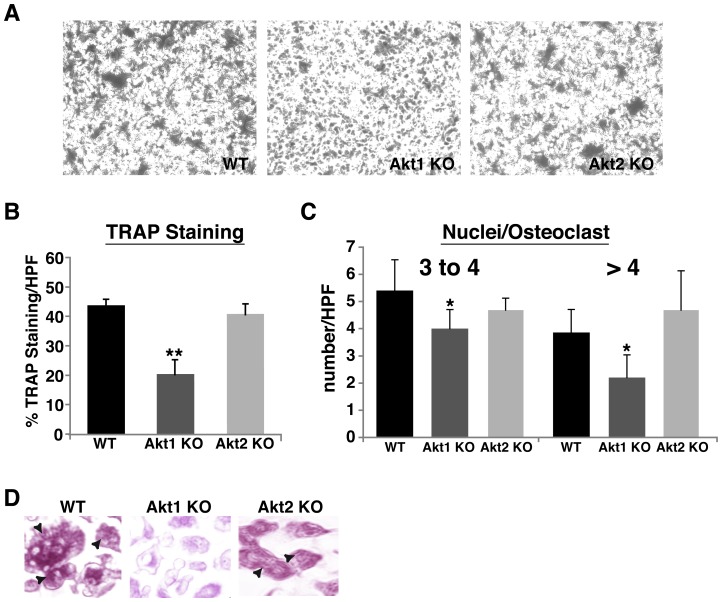
Reduced osteoclast formation in Akt1-deficient mice. Bone marrow macrophages were isolated from 20-week mice of the indicated genotypes, and incubated in osteoclastogenic medium for 6 days as described in ‘Materials and Methods’. Differentiating osteoclasts from WT mice derived from matings of heterozygous Akt1 and Akt2 deficient mice gave identical results and have been combined in sections **B–D**. **A**. Representative images of TRAP staining to assess formation of multinucleated osteoclasts on day 7 (40× magnification). **B**. Percentage of TRAP positive cells expressed as area per microscopic field (200× magnification, mean ± S.D. of 6 fields, ** - p<0.01 vs. Akt WT). **C**. Quantitative assessment of the number of multinucleated TRAP positive osteoclasts on day 7 per microscopic field (200× magnification, mean ± S.D. of 6 experiments, * - p<0.05 vs. Akt1 WT). **D**. Representative examples (200× magnification) of osteoclasts with >4 nuclei (arrowheads) and showing that osteoclasts from Akt1 deficient mice have less TRAP staining than osteoclasts derived from other genotypes.

Results are presented as mean ± SEM. Statistical significance was calculated by either one-way ANOVA with Dunnett's multiple comparison test (Table 1, Figs. 1–5), by unpaired Student's t-test when two groups were compared (for Akt2 wild type vs. Akt2 knockout mice in Table 1 and Figs. 1–5), or by paired Student's t-test for cell culture studies (Figs. 6–9). P values are listed in the Figure Legends, with p≤0.01 being the minimal level of significance for ANOVA, and p<0.05 for pairwise comparisons. Graph pad Prism version 5 (GraphPad Software, Inc., LaJolla, CA) was used for the analysis.

**Table 1:** Somatic Features of Akt-deficient Mice.

	Male			Female			Male		Female	
Parameter	*Akt1^+/+^*	*Akt1^+/−^*	*Akt1^−/−^*	*Akt1^+/+^*	*Akt1^+/−^*	*Akt1^−/−^*	*Akt2^+/+^*	*Akt2^−/−^*	*Akt2^+/+^*	*Akt2^−/−^*
Weight (g)	28.23±0.44	28.74±0.48	25.28±0.61**	23.22±0.55	21.74 ± 0.38*	21.38±0.33*	27.84±0.48	28.15±0.53	23.40±0.57	21.94±0.76
Length (cm)	9.80±0.05	9.80±0.04	9.68±0.06*	9.58±0.08	9.38 ± 0.16	9.30±0.06*	9.90±0.11	9.94±0.09	9.55±0.05	9.32±0.04
Femur length (mm)	15.72±0.1	15.84±0.05	15.57 ± 0.04	15.68±0.4	15.45 ± 0.09	15.46±0.42	15.78±0.04	15.70±0.04	15.84±0.14	15.52±0.11
Whole Body BMD (g/cm^2^)	51.87±0.54	50.83±0.76	49.27 ± 0.66*	50.68±0.55	50.31±0.43	47.85±0.89*	52.72±0.51	52.78±0.48	53.86±1.14	51.60±0.86

All analyses performed between 20–22 weeks of age; number of mice studied: 7–20 per group.

*- p<0.01 vs. *Akt1^+/+^*;

**- p<0.001 vs. *Akt1^+/+^*; no comparisons among Akt2 mice were statistically significant.

## Results

### Akt1 deficiency leads to diminished somatic growth and reduced bone mineral density

Previous studies have shown that mice lacking Akt1 have both embryonic and postnatal growth defects [29]–[31], while most reports have found that loss of Akt2 did not impair somatic growth [32], [33]. We have confirmed these findings in a cohort of Akt knockout mice in the C57/BL6 background bred at our institution. At ∼20 weeks of age, mice with Akt1 deficiency weighed 90% (male) or 92% (female) as much as wild-type controls from the same litters (Table 1). In contrast, at this age both male and female Akt2-deficient mice were of normal weight (Table 1).

We assessed whole-body bone mineral density (BMD) in age-matched ∼20-week wild type and Akt-deficient animals by peripheral dual energy X-ray absorptiometry. Whole-body BMD was reduced by 5–6% in male and female mice lacking Akt1, but was not decreased in Akt2-deficient mice of either gender (Table 1).

### Decreased long bone strength and mass in mice lacking one or two Akt1 alleles

We tested bone strength by examining resistance to fracture in three-point bending assays on isolated femurs from 20-week old animals using a high-resolution materials test apparatus. In both homozygous and heterozygous Akt1-deficient male mice we found a 15–19% reduction in failure load compared with controls (Fig. 1A). By contrast, there was no effect of Akt2 deficiency on femoral strength in comparison with wild-type sibling controls (Fig. 1A). Failure load was decreased in wild-type male mice derived from Akt2 heterozygous parents compared with wild-type male offspring of Akt1 heterozygotes (Fig. 1A), even though both Akt-deficient lines had been maintained on a C57BL/6 background and shared similar environmental conditions. This variability raises the possibility of genetic drift between the lines of Akt-haplo-deficient mice during their inbreeding, as has been described recently for rat strains [34], [35]. In contrast to results seen in male mice, loss of either Akt did not reduce failure load in females (Fig. 1B).

As bone strength is a function of both BMD and cortical and trabecular micro-architecture [36], we analyzed the organization of femurs from 20-week old mice using high-resolution micro-computed tomography (micro-CT). Measurements of cortical bone at femoral mid-shaft, including thickness, volume, and BMD were reduced in male Akt1-deficient mice (by 11–29% vs. controls depending on the parameter, Fig. 2). Most of these parameters also were significantly diminished in male Akt1 heterozygotes (by 7–17%), but except for femoral thickness were unchanged in male Akt2 knockout mice compared either with wild-type littermate controls or with wild-type mice derived from Akt1 heterozygote breeding pairs (Fig. 2). There were no statistically significant differences in cortical porosity among the genotypes (Akt1 WT, 0.67±0.06%, Akt1^+/−^, 0.66±0.02%, Akt1^−/−^, 0.65±0.07%, Akt2 WT, 0.62±0.02%, Akt2^−/−^, 0.62±0.04%). In female mice loss of Akt1 but not Akt2 significantly reduced femoral cortical thickness, volume, and BMD, but unlike in males a single Akt1 allele was sufficient to maintain normal micro-architecture (Fig. 3). As in males, cortical porosity was unaffected by Akt deficiency in females (Akt1 WT, 0.53±0.03%, Akt1^+/−^, 0.58±0.03%, Akt1^−/−^, 0.55±0.06%, Akt2 WT, 0.55±0.03%, Akt2^−/−^, 0.53±0.02%).

Trabecular bone parameters also were diminished in male mice lacking Akt1 and in heterozygotes, as seen from results of analysis by high-resolution micro-CT of the distal femoral metaphysis (Fig. 4). Reduced trabecular thickness and BV/TV also were seen in male Akt2-deficient mice and their wild-type littermates compared with male wild-type mice from comparable Akt1-deficient breeding pairs (Fig. 4), but there was no further decline in femoral trabecular bone as a result of Akt2 deficiency (Fig. 4B–E). In female mice by contrast the impact of loss of Akt1 or Akt2 on trabecular bone was minimal, as only trabecular thickness was reduced (Fig. 5). Taken together, the observations in Figs. 1–5 demonstrate a substantial contribution of Akt1 to mechanical and architectural properties of the femur, particularly in male mice, as even in haplo-deficiency measurable defects were observed. Moreover, these data indicate that Akt2 cannot compensate for loss of Akt1 in building or maintaining cortical or trabecular bone, at least within the femur.

### Reduced growth of osteoblast precursors from Akt1-deficient mice

To assess potential defects in bone-forming potential, we isolated bone marrow stromal cells (MSCs) from femurs and pre-osteoblasts from neonatal calvariae of wild type and homozygous Akt-deficient male mice, and studied their growth and osteogenic differentiation in short-term primary culture. MSCs from mice lacking Akt1 exhibited a significantly slower growth rate in serum-containing medium than wild type sibling control cells that was measurable within 48 hr, and reached a nadir of 70% of control cell numbers by 96 hr (Fig. 6B). In contrast, comparable growth rates were observed in Akt2-deficient and wild type MSCs (Fig. 7B), and in neonatal calvarial pre-osteoblasts from both Akt1- and Akt2-deficient mice (Fig. 8B, and data not shown).

We also evaluated the effects of absence of Akt1 or Akt2 on BMP2-mediated osteogenic differentiation. Loss of either Akt did not lead to up-regulation of the other mRNA or protein, and individual Akt protein expression was constant during a 10-day incubation in osteogenic medium (OM) with BMP2 in both wild type control and knockout MSCs (Figs. 6C and D, 7C). Genetic loss of either Akt1 or Akt2 did not prevent or slow osteogenic differentiation in cell culture in response to BMP2, as demonstrated by comparable production of Runx2, induction of alkaline phosphatase (AP) activity, and mineralization of osteoblasts derived from Akt1 or Akt2 knockout and wild type MSCs (Figs. 6E, 7D and E). A similar rate and extent of osteogenic differentiation also was observed in calvarial pre-osteoblasts from Akt1-deficient and wild type mice (Fig. 8). Thus, based on results pictured in Figs 6–8, we conclude that neither Akt 1 nor Akt2 is required for osteoblast differentiation of precursors isolated from long bones, but that loss of Akt1 reduces pre-osteoblast proliferation in MSCs but not in calvarial cells.

### Defective osteoclast differentiation in Akt1 deficient bone marrow macrophages

We next evaluated osteoclast differentiation using adherent bone marrow macrophages from wild type and Akt-deficient male mice as progenitors. Proliferation of precursors from each genotype in serum containing medium was comparable (data not shown). Control macrophages and cells from mice lacking Akt2 differentiated to the same extent into multinucleated tartrate-resistant acid phosphatase (TRAP) positive osteoclasts after 6 days in medium containing the osteoclastogenic growth factors, m-CSF and RANKL (Fig. 9). In contrast, osteoclast development was diminished in bone marrow macrophages from Akt1-deficient mice, as indicated by a 55% decline in TRAP positive area (Fig. 9A, B), and a 40% decrease in multinucleated cells compared with controls (Fig. 9C, D).

## Discussion

Multiple studies during the past decade have demonstrated critical roles for members of the Akt family of signaling enzymes in growth, metabolism, and tissue homeostasis (reviewed in [18], [37]–[40]. Many of the observations leading to these conclusions were made using mice engineered to have global deficiency in one of more Akts [17], [29], [31]–[33], [41], [42]. Remarkably, since mice lacking a single Akt are viable, it appears that the other two Akts are functionally able to compensate to a significant degree for loss of the third member of the family [29], [31]–[33], [42].

Regarding the skeleton, previous animal studies have led to several insights on the importance of Akt actions in bone, but have not attributed specific functions to either Akt1 or Akt2. For example, enhanced Akt activity by targeted loss of the phosphatase Pten in osteoblast precursors was found to cause increased skeletal mass in mice by stimulating bone formation [22], and global deficiency of both Akt1 and Akt2 led a severe bone deficiency phenotype, although these mice had other major defects in tissue developmental programs, and died in the perinatal period [17]. Here we have studied the effects of Akt deficiency on long bones in mature mice by performing a detailed anatomic, biochemical, and functional analysis on the femur in C57BL/6 mice lacking either Akt1 or Akt2. We found that loss of Akt1 substantially altered both cortical and trabecular micro-architecture and reduced femoral strength but that lack of Akt2 had little impact on bone structure or function.

### Diminished BMD and reduced fracture resistance in Akt1 deficiency

Our key findings are that loss of Akt1 profoundly reduced BMD and bone strength in femurs from adult mice, with both defects being more significant in males, where heterozygous Akt1 deficiency also led to structural and functional deficiencies (Table 1, Figs. 1–3). Other femoral micro-architectural parameters also were decreased in the absence of Akt1, particularly in male mice, with deficits being observed in cortical thickness and volume, and in trabecular spacing, thickness, number, and BV/TV (Figs. 2–5). Our results extend the more limited data obtained with juvenile Akt1-deficient male mice studied at 8-weeks of age [30], a time prior to the maturation of the skeleton [43], in which loss of Akt1 was associated with smaller decreases in femoral BMD than found here and with minimal alterations in cortical bone [30].

Why are the skeletal deficiencies in mice lacking Akt1 more significant in males than in females? One possibility is that there are collaborative interactions between Akt1-mediated signaling cascades and the actions of the male-enriched hormones, androgens, on bone. Androgens such as testosterone enhance trabecular and periosteal bone formation [44]–[46], with the latter effects being regulated in part after conversion of androgens to estrogens [44]. On a cellular level, androgens promote proliferation of osteoblast progenitors and their subsequent differentiation [46], in part through local production of growth factors, such as IGFs [46], which act via tyrosine kinase receptors to stimulate the PI3-kinase – Akt signaling pathway [47]. Thus, when Akt1 levels are reduced or absent, androgen effects on bone could be attenuated.

There has been little information on the actions of Akt2 on the skeleton. We now show that by itself Akt2 deficiency appears to have little impact on bone. Compared with wild-type sibling controls, there were no reductions in total body BMD in adults of either gender, a modest decline in femoral BMD in males, and no alterations in femoral strength in either males or females (Fig. 1–5). We thus conclude from our studies that the role of Akt2 in the skeleton is modest, at least under circumstances in which Akt1 continues to be expressed normally.

We did find that failure load and trabecular bone thickness and BV/TV were decreased in wild-type male mice derived from Akt2 heterozygous parents compared with wild-type male offspring of Akt1 heterozygotes (Fig. 1A, Fig. 4), an observation that is potentially puzzling, since both Akt-deficient lines had been maintained on a C57BL/6 background [29], [32], and at least in our breeding colony, shared similar environmental conditions. This variability raises the possibility of genetic drift between the lines of Akt-haplo-deficient mice during their inbreeding for multiple generations, as has been described recently for rat strains [34], [35], and will need further investigation to assess.

### Reduced osteoblast growth in the absence of Akt1

The decrease in femoral strength and BMD, and the reduction in other long bone parameters in mice lacking Akt1 could be caused by a decline in the number of osteoblasts, which could derive from reduced proliferation and/or diminished survival, or by a decrease in the rate or extent of osteogenic differentiation. We find that long bone MSCs in primary culture from Akt1-deficient male mice have a major growth defect, as the increase in cell number was diminished compared with wild-type controls, but when studied at equivalent densities, both groups underwent normal differentiation (Fig. 6). By contrast, both osteoblast growth and differentiation were normal in MSCs from Akt2-deficient mice (Fig. 7). Surprisingly, growth defects were not observed in osteoblast progenitors isolated from neonatal calvariae of male mice lacking Akt1 (Fig. 8), which is in agreement with previous studies [30]. In addition, we did not note any increases in cell death in culture regardless of the Akt genotype (data not shown), although a rise in calvarial osteoblast apoptosis in the absence of Akt1 has been reported [30]. Taken together, our results suggest major effects of Akt1 on the proliferative capabilities of osteoblast progenitors derived from long bones but not on calvarial pre-osteoblasts, indicating intrinsic differences among osteoblast populations in their responses to signaling pathways. Further study with osteogenic precursors isolated from mice with cell-type specific Akt1 knockouts will be needed to test this hypothesis and to define the relevant biochemical and molecular mechanisms.

### Reduced osteoclast differentiation in the absence of Akt1

Osteoclastogenesis also was impaired in the absence of Akt1 in adherent bone marrow macrophages isolated from long bones, as evidenced by 40–50% declines in the extent of TRAP staining and in the number of multinucleated cells versus controls, but was normal in progenitors from Akt2-deficient mice (Fig. 9). Although previous studies employing acute knockdown of Akt1 and Akt2 suggested that each Akt contributed to osteoclast differentiation [48], our observations support a more specific role for Akt1 in osteoclastogenesis [30], [49]. Based on our *in vitro* data, we envision that histomorphometric studies would demonstrate reduced osteoclast activity in Akt1-deficient mice, and when coupled with our observations on decreased proliferation of long bone osteoblast precursors from male mice lacking Akt1 (Fig. 6), support the hypothesis that the bone phenotype of Akt1 deficiency resembles ‘low-turnover’ osteopenia [3].

### Summary and perspective

Our results demonstrate differential actions of Akt1 and Akt2 on skeletal growth and maturation in mice. Genetic loss of Akt1 throughout life reduced whole body and femoral BMD when measured at 20 weeks of age, and was responsible for diminished femoral strength, particularly in males. Cell-based studies suggested that Akt1 deficiency was deleterious to both osteoblast and osteoclast precursor development, but by different mechanisms. In contrast, loss of Akt2 exerted substantially smaller effects on overall bone strength and micro-architecture that were not manifested by alterations in either osteoblast or osteoclast proliferation or differentiation. As osteoblast Akt1 also has been shown to positively regulate coupled osteoclastogenesis [50], it is clear that Akt1 exerts facilitating effects on the two key lineages responsible for promoting normal bone development and for ensuring normal bone integrity and strength. Further studies with osteoblast- and osteoclast-specific knockouts of Akt1, along with the identification and analysis of critical regulatory pathways, are needed to translate these fundamental observations into insights that are applicable to the study and treatment of human skeletal disorders.
